# Gene expression profiling by cDNA-AFLP reveals potential candidate genes for partial resistance of ‘Président Roulin’ against *Venturia inaequalis*

**DOI:** 10.1186/1471-2164-15-1043

**Published:** 2014-11-29

**Authors:** Héloïse Bastiaanse, Yordan Muhovski, Olivier Parisi, Roberta Paris, Dominique Mingeot, Marc Lateur

**Affiliations:** Life Sciences Department, Breeding and Biodiversity Unit, Walloon Agricultural Research Center, Rue de Liroux, 4, 5030 Gembloux, Belgium; Life Sciences Department, Bioengineering Unit, Walloon Agricultural Research Center, Chaussée de Charleroi, 234, 5030 Gembloux, Belgium; Consiglio per la Ricerca e la sperimentazione in Agricoltura, Centro di Ricerca per le Colture Industriali, CRA-CIN, via di Corticella 133, 40128 Bologna, Italy; Plant Pathology Unit, Gembloux Agro-Bio Tech, University of Liège, Passage des déportés 2, 5030 Gembloux, Belgium

**Keywords:** cDNA-AFLP, Partial resistance, Apple scab, *Venturia inaequalis*

## Abstract

**Background:**

Scab, caused by the fungus *Venturia inaequalis*, is one of the most important diseases of cultivated apple. While a few scab resistance genes (R genes) governing qualitative resistance have been isolated and characterized, the biological roles of genes governing quantitative resistance, supposed to be more durable, are still unknown. This study aims to investigate the molecular mechanisms involved in the partial resistance of the old Belgian apple cultivar ‘Président Roulin’ against *V. inaequalis*.

**Results:**

A global gene expression analysis was conducted in ‘Président Roulin’ (partially resistant) and in ‘Gala’ (susceptible) challenged by *V. inaequalis* by using the cDNA-AFLP method (cDNA-Amplified Fragment Length Polymorphism). Transcriptome analysis revealed significant modulation (up- or down-regulation) of 281 out of approximately 20,500 transcript derived fragments (TDFs) in ‘Président Roulin’ 48 hours after inoculation. Sequence annotation revealed similarities to several genes encoding for proteins belonging to the NBS-LRR and LRR-RLK classes of plant R genes and to other defense-related proteins. Differentially expressed genes were sorted into functional categories according to their gene ontology annotation and this expression signature was compared to published apple cDNA libraries by Gene Enrichment Analysis. The first comparison was made with two cDNA libraries from *Malus* x *domestica* uninfected leaves, and revealed in both libraries a signature of enhanced expression in ‘Président Roulin’ of genes involved in response to stress and photosynthesis. In the second comparison, the pathogen-responsive TDFs from the partially resistant cultivar were compared to the cDNA library from inoculated leaves of *Rvi6* (*HcrVf2*)-transformed ‘Gala’ lines (complete disease resistance) and revealed both common physiological events, and notably differences in the regulation of defense response, the regulation of hydrolase activity, and response to DNA damage. TDFs were *in silico* mapped on the ‘Golden Delicious’ apple reference genome and significant co-localizations with major scab R genes, but not with quantitative trait loci (QTLs) for scab resistance nor resistance gene analogues (RGAs) were found.

**Conclusions:**

This study highlights possible candidate genes that may play a role in the partial scab resistance mechanisms of ‘Président Roulin’ and increase our understanding of the molecular mechanisms involved in the partial resistance against apple scab.

**Electronic supplementary material:**

The online version of this article (doi:10.1186/1471-2164-15-1043) contains supplementary material, which is available to authorized users.

## Background

Apple scab caused by the hemi-biotrophic ascomycete *Venturia inaequalis* (Cke.) Wint. is one of the most serious diseases of apple (*Malus* x *domestica*, Borkh.) worldwide, causing huge economic losses. Scab infection leads to deformation in the shape and size of fruits, premature leaf and fruit drop, and enhances susceptibility of the tree to chilling and freezing injuries [1]. Currently, multiple applications of fungicides are required for effective control in commercial orchards planted with susceptible cultivars. Depending on the year and region, as many as 18 to 29 fungicide treatments may be necessary in one season to control the disease. For apple orchards in France, pesticide treatments costs account for about 10% of the fixed production expenses, representing a substantial cost per kg of apple (0.031 €) [2]. This intensive use of fungicides in orchards raises ecological problems and human health concerns in addition to the economic cost.

An effective alternative to chemical control is the use of scab-resistant apple cultivars. Phenotypically, the effect of resistance genes against *V. inaequalis* has been showed to cover a continuum from complete immunity to near-susceptibility depending on genetic background, pathogen and environment [3]. Despite the great deal of gray area between the extremes [4] hampering the classification of some genotypes, apple scab resistance is often qualified either as complete, when the pathogen growth is fully inhibited (complete or qualitative resistance), or partial, when the resistance allows limited but significantly reduced pathogen growth as compared to a susceptible genotype (partial or quantitative resistance). Based upon the extent of pathogen growth and nature of symptoms, Chevalier et al. [5] classified the macroscopic foliar symptoms into four classes; classes from 0 to 3 are categorized as resistance responses while class 4 is considered to be a susceptible response. It is usually referred that complete resistance is determined by major resistance gene (R gene) while incomplete resistance involved multiple genes or loci of partial effect (QTLs, Quantitative Trait Loci).

R genes, typically providing high levels of resistance, are relatively easy to manipulate. For these reasons, they were extensively used in both basic research and applied breeding programs. To date, at least 17 major scab resistance genes have been identified and mapped across nine linkage groups of the apple genome [3]. For more than 50 years, one of these R genes, the *Rvi6* (*Vf*) gene from *Malus floribunda* 821, has provided effective resistance against apple scab by allowing a reduction of 75% in the number of fungicide treatments [6]. Nevertheless, the use of single R gene-mediated resistance for crop protection is hampered by a lack of durability, particularly with pathogens having high evolutionary potential, as with *V. inaequalis*[7]. This ephemeral nature of R gene-mediated resistance is highlighted by the recent emergence of some races of *V. inaequalis* that are virulent on cultivars carrying the widely-deployed *Rvi6* (*Vf*) gene [8]. In contrast to major genes, the performance of partially resistant cultivars in the orchard is a function of the gene effects and spectra, which is thought to be more durable than single R gene-mediated resistance [9]. This durability could be explained by the smaller effects of partial resistance that result in a lower selection pressure on the pathogen and/or its presumed broader spectrum. Also, because partial resistance is controlled by multiple genes, pathogen isolates that overcome one of the genes would gain only a marginal advantage [4]. In apple, partial resistance has been mapped as QTLs to 10 out of the 17 linkage groups of the genome [10–14].

Extensive efforts have been made to clone and characterize major scab R genes, and the downstream responses that they trigger have become better understood. R genes typically encode proteins that recognize pathogen effectors or modifications of plant proteins that are targets of those effectors [4]. In this respect, the *Rvi6* (*Vf*) resistance locus revealed the presence of a cluster of four resistance gene paralogs (called *HcrVf* genes), similar to the tomato *Cf* resistance gene, encoding leucine-rich repeats receptor-like proteins (LRR-RLP) [15] and the *Rvi15* (*Vr2*) was reported to contain three TIR-NBS-LRR genes [16]. The function of all these genes was analyzed and only two of them, *HcrVf2*[17, 18] and *Vr2-C*[19] for *Rvi6* and *Rvi15*, respectively, were proven to be functional against *V. inaequalis*. Literature is not in agreement on *Vf1a* (syn. *HcrVf1*) function [18, 20]. Recognition of *V. inaequalis* by these proteins triggers downstream defense reactions involving putative LRR receptor-like protein kinases [21, 22] and several defense-related proteins, such as b-1,3-glucanase, ribonuclease-like PR-10, cysteine protease inhibitor, endochitinase, ferrochelatase, and ADP-ribosylation factor [23, 24]. Methallothionein may also play a role in plant defense against *V. inaequalis* as it is present in large amounts in the apoplast of the *Rvi6* cultivar ‘Remo’. Finally, recent publications explored the network of defense response triggered in *Rvi6* (*HcrVf2*)-transformed apple plants using wide genome expression techniques, such as the PCR-based suppression subtractive hybridization and the dHPLC for cDNA- Amplified Fragment Length Polymorphism (cDNA-AFLP) transcript profiling [25, 26]. These studies gave new insight into the understanding of the plant pathogen-interaction that results in complete scab resistance. Nevertheless, the function of genes underlying the QTLs for partial apple scab resistance remains unknown. They are believed not to be based on pathogen recognition systems, as it is the case with most major R genes, but the possibility cannot be excluded [27].

The partially resistant apple cultivar ‘Président Roulin’ is an old Belgian cultivar that is used in apple breeding programs of the Walloon Agricultural Research Center (CRA-W) to broaden genetic apple scab resistance, and therefore reduce the risk of resistance breakdown. Under heavy scab infection, this cultivar shows typical resistance symptoms of chlorosis and necrosis with only slight sporulation (Chevalier class 3a). Its resistance against *V. inaequalis* has been durable for over 25 years in scab evaluation in different orchards of the CRA-W in Belgium without any fungicide treatment [28]. The partial resistance has been shown to be polygenic, but highly heritable [9].

In this study we investigated the defense response of ‘Président Roulin’ by identifying genes differentially expressed between this cultivar and the susceptible ‘Gala’ cultivar after pathogen challenge. For this purpose, cDNA-AFLP technology was chosen as it allowed a survey of transcriptional changes with no prior assumptions about which genes might be involved in the plant response [29]. This technique constitutes a robust solution for differential display, detecting changes in gene expression between samples, and it has been successfully applied in several quantitative expression studies in apple [26, 30–33]. The genes identified in this study were annotated and sorted into Gene Ontology (GO) categories. Comparisons were then made with various *Malus* x *domestica* libraries: two EST libraries from uninfected young expanding leaves [34, 35] and a cDNA library from an apple susceptible line transformed with the major scab R gene *Rvi6* (*HcrVf2*) [25]. Common as well as different defense pathways were revealed and are discussed. Finally, we checked for co-localizations of our differentially expressed genes with resistance gene analogues (RGAs), QTLs and mapped R genes for apple scab resistance published in the literature.

## Results

### Fungal development across post inoculation time points

**Figure 1:**
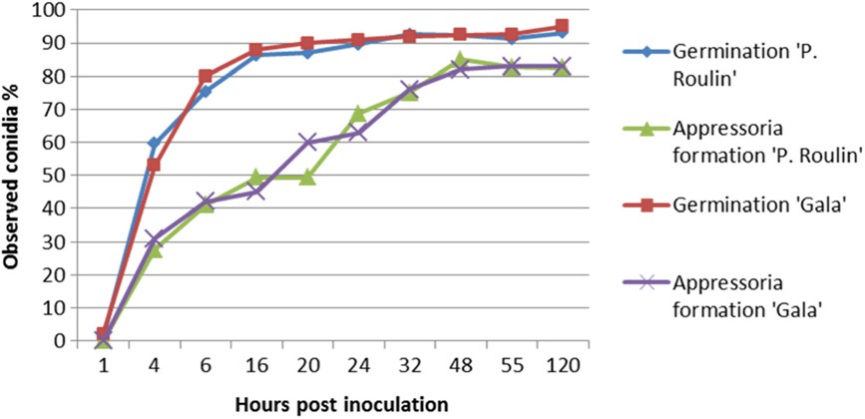
**Kinetics of** ***V. inaequalis*** **conidial development on ‘Président Roulin’ and ‘Gala’ leaves.** Germination of conidia and formation of appressoria were observed under light microscopy over time post inoculation. Fungal tissues were stained on whole leaves with periodic acid-basic fuchsin according to the method of Preece [97].

Microscopic observations revealed no significant difference between ‘Président Roulin’ (partially resistant) and ‘Gala’ (susceptible) for pathogen development at the early stages of infection. This comprises conidia germination, formation of appressoria and cuticle penetration (Figure 1). On both cultivars, conidia germination began within 4 hours post inoculation (hpi), and at 16 hpi most of the conidia had produced germ tubes. At this time, the rudimentary germ tubes had bulged at the tip to form characteristic appressoria adhering to the cuticle. At the end of the 24 hour period, the process was further advanced. Shortly after the formation of the appressoria, infection hyphae appeared and penetrated the host. At 48 hpi, 80% of the appressoria were formed and invasion of the host plant started with the expansion of the primary stroma. With the staining method used in this study, the subcuticular growth of the fungus was difficult to observe because it was poorly stained. Nevertheless, we can assume that, when the stroma was visible, at 120 hpi, subcuticular development on the susceptible host, ‘Gala’, had significantly exceeded that of the partially resistant ‘Président Roulin’ (data not shown). This difference in the extent of colonization of the fungus between the two cultivars remained throughout the whole infection cycle of the fungus. Between 7 and 12 days after inoculation, apple scab symptoms became macroscopically visible on ‘Président Roulin’ and ‘Gala’. After 21 days, 90% of the leaf surface of ‘Gala’ (susceptible) was covered by sporulating apple scab lesions (class 4) [5]. However, typical chlorotic and necrotic lesions with slight sporulation were observed on leaves of ‘Président Roulin’ (partially resistant), covering less than 15% of the leaf surface. These symptoms were considered as resistance responses and were classified in the class 3b, as described by Chevalier et al. [5].

### cDNA-AFLP fingerprints: optimization and identification of differentially expressed transcripts

We used the AFLPinSilico application to choose the optimal restriction enzyme combination for the cDNA-AFLP experiments. The results of the different parameters for each enzyme pair combination are shown in Table 1. Enzymes with 4- or 5-base recognition sites yielded the highest number of Transcript Derived Fragments (TDFs), although these were generally relatively short and highly redundant, with up to 5 cleavage sites per cDNA. In opposite, the *Eco*RI/*Mse*I recognized cleavage sites on less than half (34%) of the apple full-length cDNA tested, but this combination of restriction enzymes generated more informative TDFs than all the other enzyme pairs tested. In fact, *Eco*RI provided the longest TDF (mean size of 234 bp) with the lowest redundancy (0.5 restriction sites per cDNA) and derived at least partially from coding regions (683 bp from the poly(A) + tail). This enzyme was therefore the most appropriate and it was chosen in combination with *Mse*I for cDNA-AFLP analysis.

**Table 1:** **Suitability of restriction enzymes for use in cDNA-AFLP analysis in apple**

Enzyme ^a^	Restriction site	TDF visualized ^b^	N° cleavage sites	Position (bp) ^c^	Length (bp) ^d^
**SAU3A**	GATC	69%	5.5	723	133
**TAQI**	TCGA	67%	4.5	680	147
**DDEI**	CTNAG	70%	4.5	650	156
**ECORII**	CCWGG	66%	2.0	605	222
**APOI**	RAATTY	59%	2.4	676	167
**ECORI**	GAATTC	34%	0.5	683	234

^a^450 Full Length cDNA from *Malus* x *domestica* were analyzed *in silico* for patterns of cleavage by different restriction enzymes in combination with *Mse*I.

^b^Percentage of cDNAs that yielded a TDF of a size that could be resolved on a 5% polyacrylamide gel (between 50 and 1000 bp) after cleavage with the particular enzyme in combination with *Mse*I.

^c^Average distance between the last recognition site and the polyadenylation site.

^d^Mean size of restriction fragments, expressed in base pairs.

**Figure 2:**
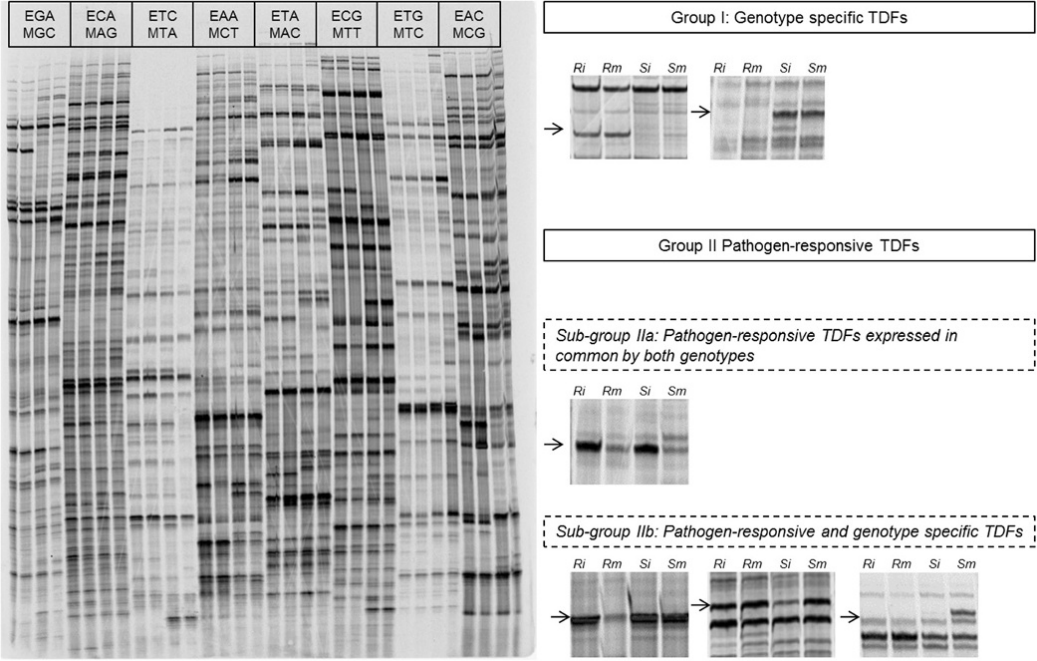
**Expression patterns of apple genes displayed by cDNA-AFLP fingerprints.** The cDNA-AFLP compares transcriptional profiles from ‘Président Roulin’ (partially resistant) and ‘Gala’ (susceptible) mock-inoculated or challenged by *V. inaequalis* at 48 hpi. The 32 samples are arranged in 8 groups according to the different specific primers tested during the selective amplification step of the AFLP procedure. E and M refer to the *Eco*RI and *Mse*I primers, followed by the selective nucleotides used. Within each of the 8 groups samples are ordered as follows: ‘Président Roulin’ infected (Ri) and mock-inoculated (Rm), and ‘Gala’ infected (Si) and mock-inoculated (Sm). Differentially expressed TDFs were classified into 2 categories: genotype-specific TDFs (group I) and pathogen-responsive TDFs (group II), further divided into two sub-groups; pathogen-responsive TDFs expressed in common by both genotypes (sub-group IIa) and pathogen-responsive and genotype specific TDFs (sub-group IIb). Illustrations are given.

The cDNA-AFLP analysis using 141 primer combinations in ‘Président Roulin’ and ‘Gala’ resulted in 30 to 100 TDFs per primer combination, depending on the number of additional bases used for the selective amplification step, and a total of about 10,250 TDFs in each cultivar (representing a total of 20,500 for both cultivars). TDFs ranged in size from 30 to 800 bp. Figure 2 shows a typical cDNA-AFLP profile of the two apple genotypes challenged by *V. inaequalis* or water. Considering that 123 primer pairs with two additional selective nucleotides (*Eco*RI + 2/*Mse*I + 2) were tested out of the 256 possible primer pair combinations, and taking into account that about 40% of the apple cDNA could potentially be visualized with the restriction enzyme *Eco*RI and *Mse*I, we estimated that we analyzed a representative sample of approximatively 19% of the apple genes expressed in the tissues.

At the stringent threshold used, only 4.6% of all the 20,500 generated fragments exhibited significant differences in intensity among the different genotypes or treatments. According to their banding patterns, the differentially expressed TDFs were classified into two groups: (I) genotype-specific TDF, for all banding patterns differing between the genotypes but not affected by fungal infection and (II) pathogen-responsive TDFs representing all the TDFs showing differential expression during infection. Group II was further divided into two subgroups: (IIa) pathogen-responsive TDFs, representing the TDFs with differential expression upon pathogen challenge in both genotypes and (IIb) pathogen-responsive and genotype-specific TDFs, representing the TDFs showing differential expression induced by the pathogen in one of the two cultivars.

**Figure 3:**
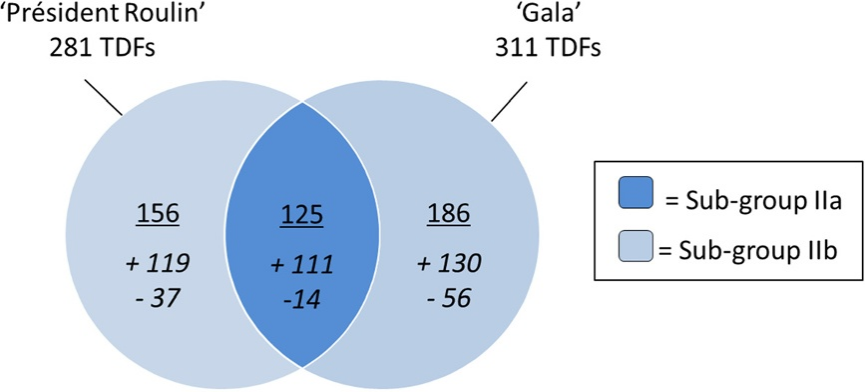
**Venn diagram showing number of pathogen-responsive TDFs in ‘Président Roulin’ (partially resistant) and/or ‘Gala’ (susceptible).** Group II of TDFs was classified into two sub-groups: pathogen-responsive TDFs expressed in common by both genotypes (sub-group IIa) and pathogen-responsive and genotype specific TDFs (sub-group IIb). ‘+’ and ‘-’ represent up- and down-regulation, respectively.

The first group (group I, genotype-specific) accounted for about 1.7% of all generated fragments (232 TDFs present only in ‘Président Roulin’ and 115 in ‘Gala’). In contrast, 281 (230 up-and 51 down-regulated) and 311 TDFs (241 up- and 70-down regulated), respectively, were identified in ‘Président Roulin’ and ‘Gala’ as significantly differentially expressed after fungal infection (Figure 3). These pathogen-responsive TDFs (group II) accounted for about 2.9% of all the 20,500 TDFs analyzed for both cultivars. Among them, 125 (111 up-regulated and 14 down-regulated) overlapped between the two genotypes (subgroup IIa). The remaining 156 and 186 TDFs, for ‘Président Roulin’ and ‘Gala’, respectively, displayed differential expression after fungal attack that depended on the genotype. They were classified into subgroup IIb (pathogen-responsive and genotype-specific) and represented about 1.7% of the fragments. From our point of view, these bands differentially expressed in ‘Président Roulin’ only, are the most interesting as they could be involved in specific plant defense reaction against *V. inaequalis*. In fact, genes that showed only a genotype effect may reflect the effect of the genetic background, whereas genes exhibiting only a treatment effect may represent a general plant response to pathogen challenge that does not determine the final different phenotype.

**Figure 4:**
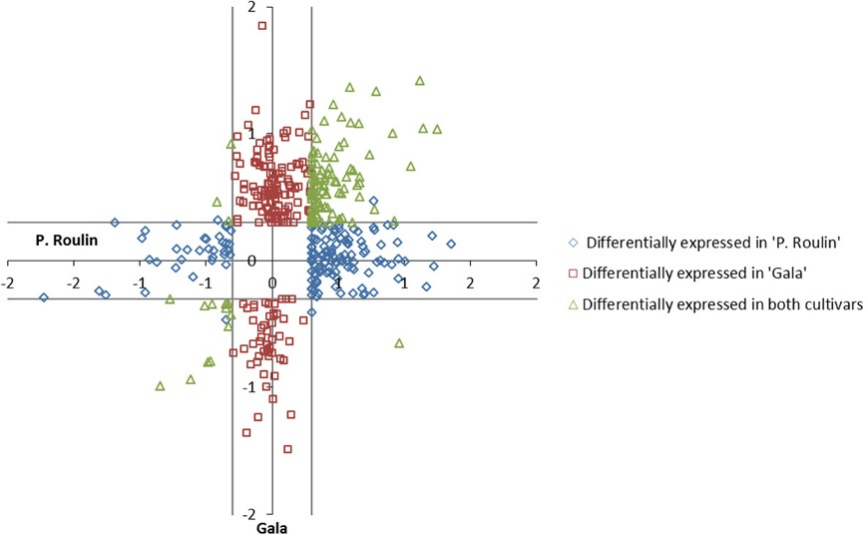
**Scatter plot of log10-gene expression fold changes of pathogen-responsive TDFs from ‘Président Roulin’ and ‘Gala’.** Fold changes are relative to mock inoculation. Colour-coded plots represent TDFs differentially regulated in one of the cultivars (cultivars-specific) or in both cultivars (common). Log ratios >0 or <0 indicate up- or down-regulation, respectively, dashed lines set at 0.3 and −0.3 (+ − log_10_2) correspond to the threshold of a two-fold change in gene expression that was used as the cut-off value for biological significance.

We then considered the amplitude and direction of expression changes for all pathogen-responsive TDFs (group II). We plotted the log10 transformed expression ratios of ‘Président Roulin’ against ‘Gala’ and distinguished the TDFs that were differentially expressed in only one of the two lines (blue squares for ‘Président Roulin’ and red squares for ‘Gala’, Figure 4) from those being differentially expressed by both cultivars (green triangle, intersect of the two circles in the Venn diagram of Figure 3). This graph showed the overall similarity and specificity of gene expression in the TDFs differentially expressed in common by ‘Président Roulin’ and ‘Gala’: most of these TDFs were differentially regulated in the same direction (up or down-regulation) by both cultivars. Fold-changes from 2 to 70 were observed, with the majority of the TDFs showing a difference in expression less than 7-fold.

### Sequences annotation

Two-hundred and fifty nine TDFs out of the 281 pathogen-responsive TDFs from the resistant cultivar and part of the genotype-specific TDFs (131 TDFs) were excised from the gels, re-amplified and cloned (390 TDFs in total). No bands belonging to the susceptible cultivar ‘Gala’ were excised and a priority was given to TDFs of sufficient length (upper part of the gel), to facilitate their functional characterization. Two clones were sequenced for each re-amplified TDF and for 38% of them (146 TDFs) a different sequence between the two clones was found, leading to a total of 536 sequences. To limit redundancy, sequences were clustered using the software Egassembler [36, 37] and then compared to the whole apple genome sequence assembly in order to identify the unigenes [38]. This returned 497 unique sequences (29 contigs and 468 singletons) from 53 bp to 803 bp that were annotated and submitted to the NCBI database with accession numbers assigned (Additional file 1).

Among the 497 sequences, 69% (344 TDFs) were similar to known expressed sequences in public databases (319 could be annotated, 25 were similar to encoded proteins with unclear function), while 153 sequences had no matches in the NCBI database. With the exception of 15 sequences similar to genes belonging to the apple stem pitting virus (ASPV), all TDFs were similar to sequences from model plants, with *Vitis vinifera*, *Ricinus communis* and *Populus trichocarpa* being the three most represented species. As expected, most TDFs (80%) were found to be similar (BLAST E-value <1e-4) to *Malus* x *domestica* sequences derived from the whole genome sequence of apple [38]. The ASPV is a widespread pathogen of pome fruit usually transmitted by the rootstock and frequently latent in the host [39]. As the cDNA encoding for ASPV coat proteins was found in both inoculated and controlled plants and as no symptoms of ASPV were observed in apple trees used for the experiments we believe that the virus was latent and did not interfere with gene expression data.

**Figure 5:**
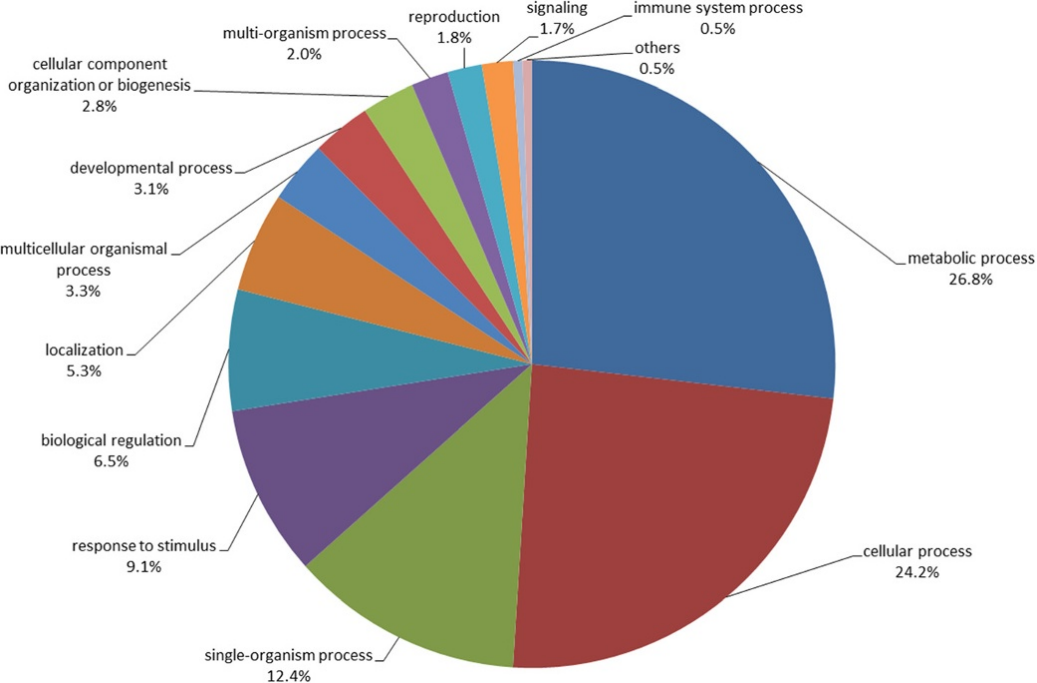
**Distribution of differentially expressed TDFs within the GO categories of biological processes.** GO annotations were made according to the International Gene Ontology Consortium using the automatic bioinformatics software Blast2GO.

Putative functions of the apple TDFs were then classified according to the GO vocabulary. Figure 5 shows the percentages of apple genes assigned to the biological processes. About 27% of the annotated sequences have metabolic roles (particularly in protein and carbohydrate metabolism) and 24% are involved in cellular processes. Other relevant groups, accounting for 12, 10 and 7% of the TDFs, respectively, include single-organism process, response to stimulus (particularly response to biotic stress, oxidative stress, response to wounding and defense response, listed in Table 2) and biological regulation. In addition to TDFs related to plant defense response according to the GO classification, we also found TDFs showing similarities with genes encoding for proteins reported to have a potential role in the general defense response pathway through careful analysis of the scientific literature. These TDFs are also potential candidate genes for partial resistance of ‘Président Roulin’ against *V. inaequalis*. These TDFs encoded proteins involved in the recognition of the pathogen, signal transduction, oxidation reduction process, photosynthesis, transport, response to environmental stress, metabolism, transcription, and cell wall organization (Table 3). Expression of TDFs of interest was further investigated by qRT-PCR and putative functions of some of these proteins in the partial resistance against *V. inaequalis* are discussed.

**Table 2:** **TDFs associated with plant defense response, response to oxidative stress and response to wounding**

TDF	Expression pattern ^a^		Annotation	GO annotations ^b^
**1AU61′**	IIb	+	e3 sumo-protein ligase siz1	P:induced systemic resistance; P:negative regulation of systemic acquired resistance
**37DU41**	IIb	+	cysteine proteinase inhibitor	P:defense response
**43CU118**	IIb	-	tmv resistance protein	P:defense response; P:innate immune response
**43DU149**	IIb	+	peroxidase	P:response to oxidative stress
**43DU149′**	IIb	+	nucleotide binding site leucine-rich repeat disease resistance protein	P:defense response
**44AU9**	IIb	+	LRR receptor kinase-like protein	P:defense response
**51HU129′**	IIb	+	tocopherol cyclase	P:regulation of defense response
**56 AU33′**	IIb	+	nbs-lrr resistance protein	P:defense response
**14GU213**	IIa	+	TMV resistance protein N-like	P:innate immune response; P:defense response
**33FU130′**	IIa	-	Avr9/Cf-9 rapidly elicited protein	P:response to wounding
**34EU#2**	IIa	+	12-oxophytodienoate reductase	P:response to wounding
**34CU81′**	IIa	+	nadp-dependent oxidoreductase	P:response to oxidative stress
**34FU145′**	IIa	+	disease resistance protein	P:defense response; P:innate immune response
**41CU29′**	IIa	+	protein bonzai 3-like	P:positive regulation of cellular defense response
**42AU1**	IIa	+	nad-dependent epimerase dehydratase	P:defense response to bacterium
**46CU57′**	IIa	+	cc-nbs-lrr resistance protein	P:defense response
**47CU77′**	IIa	+	ferredoxin-nadp + reductase	P:defense response to bacterium
**54DU58**	IIa	+	progesterone 5-beta-reductase	P:response to wounding
**55BU33**	IIa	+	multidrug resistance protein abc transporter family	P:response to wounding
**34DU#2**	I		adp-ribosylation factor gtpase-activating protein agd2-like	P:systemic acquired resistance
**43BU45**	I		proteasome subunit beta type-6	P:regulation of plant-type hypersensitive response
**43CU113′**	I		type ii peroxiredoxin	P:response to oxidative stress
**43HU225**	I		cell wall-associated hydrolase	P:response to oxidative stress
**44CU85**	I		acetylornithine aminotransferase	P:defense response to bacterium
**44HU193′**	I		disease resistance protein at3g14460-like	P:defense response
**45CU49**	I		formamidopyrimidine-dna glycosylase	P:response to oxidative stress
**52BU9**	I		nad-dependent epimerase dehydratase	P:defense response to bacterium

^a^TDF Expression pattern according to the cDNA-AFLP (induced + or repressed -) at 48 hpi by *V. inaequalis*: genotype-specific TDFs (I), pathogen-responsive TDFs expressed in common by both genotypes (IIa) and pathogen-responsive and genotype specific TDFs (IIb).

^b^GO annotations were made using the automatic bioinformatics software Blast2GO.

**Table 3:** **Gene expression analysis of selected TDFs by qRT-PCR in ‘Président Roulin’ (resistant) and ‘Gala’ (susceptible)**

TDF	Annotation (blastx)	Exp. ^a^	Fold induction/repression			
			cDNA-AFLP samples		Biological repetition	
			Resistant cv.	Susceptible cv.	Resistant cv.	Susceptible cv.
	**Defense response**					
43DU149’	cc-nbs-lrr resistance protein	IIb +	+7.9 ± 2.6*^b^	+1.9 ± 0.0	+2.8 ± 1.1	+1.4 ± 0.3
56AU33’	cc-nbs-lrr resistance protein	IIb +	+2.6 ± 0.1*	+1.5 ± 0.1	+2.9 ± 0.5	+1.3 ± 0.5
42BUHcrVf	HcrVf paralog	IIb +	+1.0 ± 0.1	−1.9 ± 0.2*	−1.2 ± 0.1	−1.2 ± 0.2
43CU118	TMV resistance protein	IIb -	+1.6 ± 0.1	+1.5 ± 0.2	-	-
44AU9	LRR receptor kinase-like protein	IIb +	+ 5.1 ± 1.7*	+1.4 ± 0.4	+2.2 ± 0.4*	+1.7 ± 0.0*
44GU169	2-cys peroxiredoxin	IIb +	+10.3 ± 0.1*	+2.2 ± 0.1*	+1.4 ± 0.5	+1.2 ± 0.0
54CU21	Phi class glutathione transferase	IIb +	+3.5 ± 0.0*	−1.2 ± 0.5	+1.1 ± 0.1	−1.5 ± 0.1
	**Signal transduction**					
2EU181	Putative MAP kinase	IIb +	+2.2 ± 0.0*	+1.4 ± 0.2	+2.1 ± 0.3*	−1.3 ± 0.0
39AU13	MAP kinase phosphatase	IIb +	+1.4 ± 0.0	+1.1 ± 0.2	−1.0 ± 0.1	−1.6 ± 0.0*
	**Transporter**					
46EU122	ABC transporter	IIb -	−2.4 ± 0.1*	+1.3 ± 0.0	−1.5 ± 0.1	+2.4 ± 0.0*
	**Oxidation reduction process**					
51DU17	Cytochrome P450	IIb +	+2.0 ± 0.3*	+1.1 ± 0.3	+1.0 ± 0.2	+1.1 ± 0.0
53DU34	Cytochrome P450	IIb -	−4.8 ± 0.0*	+1.5 ± 0.2	−1.9 ± 0.0*	−2.6 ± 0.1*
	**Photosyntesis**					
56AU5’	Uroporphyrinogen decarboxylase	IIb +	+6.5 ± 1.3*	+3.9 ± 1.1	−1.0 ± 0.0	−1.4 ± 0.2
	**Response to environmental stress**					
43DU149	Peroxidase 12	IIb +	+3.4 ± 0.2*	+1.2 ± 0.1	+4.0 ± 0.2*	+1.9 ± 0.2*
51HU129’	Tocopherol cyclase	IIb +	+8.1 ± 0.0*	+2.0 ± 0.1*	+6.9 ± 0.0*	+1.5 ± 0.0*
	**Metabolism**					
Consensus 44EU122/44EU118	Cysteine protease	IIa -	- 12.7 ± 4.5*	- 4.1 ± 1.2*	−68.8 ± 0.0*	−3.2 ± 0.3
37DU41	Cysteine protease inhibitor	IIb +	+ 2.4 ± 0.1*	+1.2 ± 0.1	+2.9 ± 0.0*	+1.4 ± 0.1
1AU61’	Sumo ligase	IIb +	+1.5 ± 0.3	+1.6 ± 0.1	-	-
56AU29	Chitinase	IIb +	+2.3 ± 0.1*	+1.4 ± 0.1	+2.7 ± 0.2*	+1.2 ± 0.1*
44GU182	Lysosomal Pro-X carboxypeptidase	IIb -	- 28.4 ± 4.9*	+ 1.4 ± 0.1	−22.0 ± 0.0*	−1.9 ± 0.1*
	**Transcription factor**					
53HU89	Zinc finger homeodomain protein1	IIb +	+10.3 ± 0.2*	+1.7 ± 0.1	+3.3 ± 0.1*	+1.8 ± 0.1*
	**Cell wall organization**					
44GU173	Pectin methylesterase inhibitor	IIb +	+3.3 ± 0.8*	+1.1 ± 0.1	+3.6 ± 0.9*	+1.2 ± 0.2*
	**Unknown functions**					
55FU102	No homology	IIa +	+3.0 ± 0.1*	+1.4 ± 0.5	+3.8 ± 0.2*	+1.3 ± 0.0
55HU125’	No homology	IIb +	+4.8 ± 0.0*	- 1.5 ± 0.1	+1.7 ± 0.0	+1.5 ± 0.2

^a^Expression pattern according to the cDNA-AFLP. Group IIa represents pathogen-responsive TDFs expressed in common by both genotypes and group IIb pathogen-responsive and genotype specific TDFs. “+” = induced and “–” = repressed TDF.

^b^Means and SD of fold induction (+) or repression (−) calculated by the ΔΔCt method applied using qRT-PCR. Significant fold changes were judged considering the following criteria: statistical significance of individual ΔCt values at *P* <0.01 (*) and biological significance at fold change ≥2.

### Gene enrichment analysis

**Figure 6:**
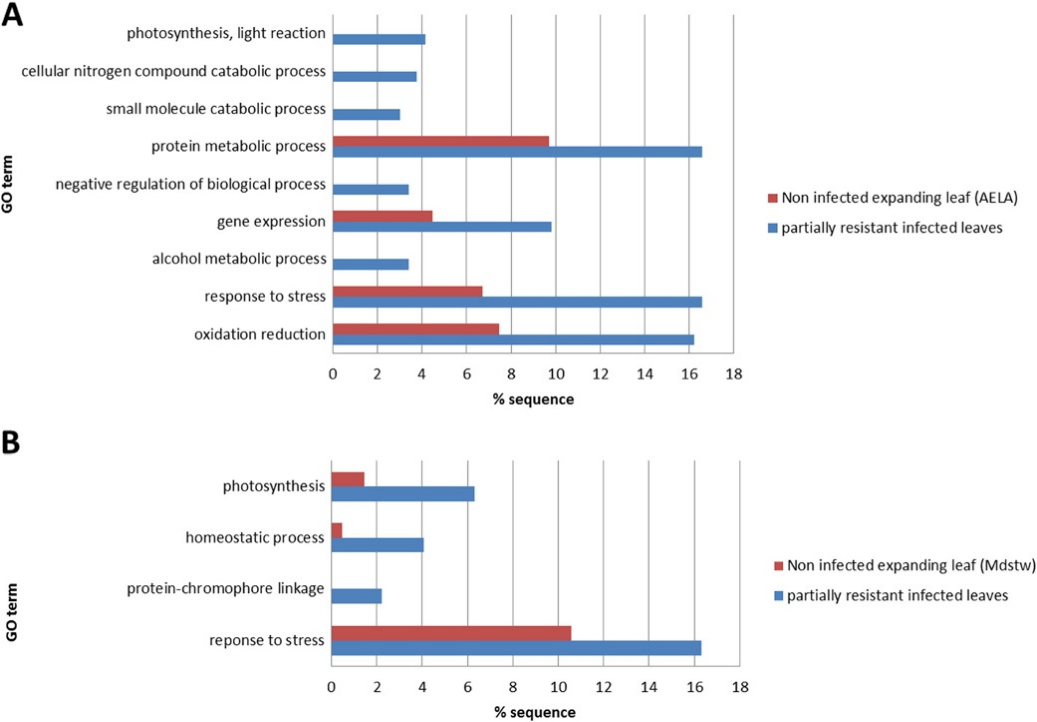
**Over-representation of GO categories in ‘Président Roulin’ cDNA-AFLP library compared to non-infected EST apple libraries.** The comparison has been made by gene enrichment analysis for Biological Process GO categories between our cDNA-AFLP library from scab-infected leaves of ‘Président Roulin’ (partially resistant) and two EST libraries from uninfected actively growing shoot of: **(A)** cultivar ‘Royal Gala’ in the library AELA [34] and **(B)** cultivar ‘Wijcik’ in the library Mdstw [35]. Gene enrichment analysis was conducted with the software Blast2Go using Fisher’s Exact Test at a p-value <0.05. No GO categories were shown to be under-represented.

The gene enrichment analysis revealed that genes accounting for the different functional GO categories were similarly represented (P > 0.05) in two subsets of our cDNA-AFLP: the TDFs being up- or down-regulated in ‘Président Roulin’ after pathogen attack (group II + and II - ). Nevertheless, when compared to two different collections of ESTs [34, 35] from uninfected expanding apple leaves (AELA and Mdstw), some biological pathways appeared to be significantly over-represented (P < 0.05) in the ‘Président Roulin’ cDNA-AFLP library (Figure 6). In both comparisons, it was notably the case for genes involved in photosynthesis and in response to stress. Additionally, other GO categories were significantly over-represented in ‘Président Roulin’ but depended on the library being compared: e.g. oxidation reduction process, regulation of gene expression in the AELA library, and homeostatic process in the Mdstw library. All these genes could be involved in the general defense response pathway of ‘Président Roulin’ against *V. inaequalis*. No biological pathways appeared to be significantly under-represented in our collection.

**Figure 7:**
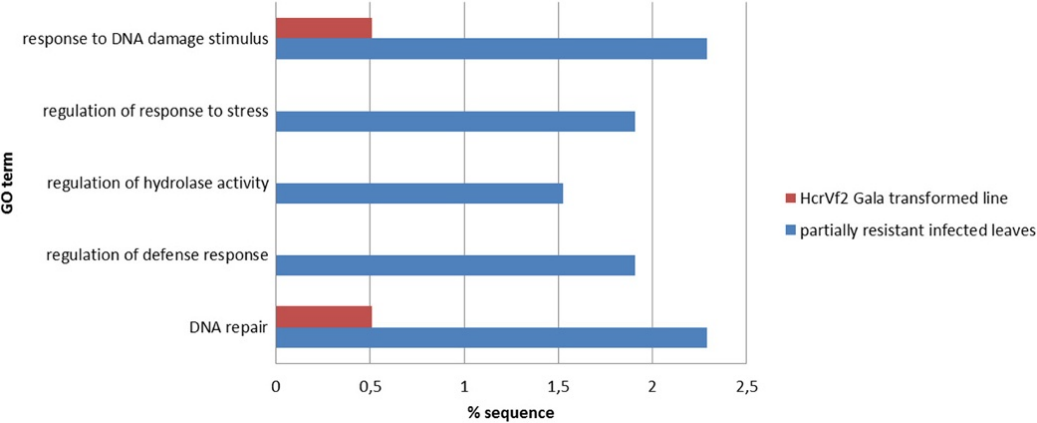
**Over-representation of GO categories in ‘Président Roulin’cDNA-AFLP library compared to** ***Rvi6*** **(** ***HcrVf2*** **)-’Gala’ transformed library.** The comparison has been made by gene enrichment analysis for the Biological Process GO category between our cDNA-AFLP library (scab-infected leaves of ‘Président Roulin’, partially resistant) compared with a cDNA library from completely resistant *Rvi6* (*HcrVf2*)-transformed ‘Gala’ lines challenged with *V. inaequalis*[25]. Gene enrichment analysis was conducted with the software Blast2Go using Fisher’s Exact Test at a p-value <0.05.

In order to assess if mechanisms leading to partial resistance against *V. inaequalis* might involve biological pathways different to a complete resistance response, we compared by gene enrichment analysis our ‘Président Roulin’ TDFs dataset to the SSH library from *Rvi6 (HcrVf2)*-transformed ‘Gala’ line challenged by *V. inaequalis*[25]. Some biological processes were over-represented in our partial resistance compared to the complete resistance mediated by *Rvi6* (*HcrVf2*) gene (Figure 7, Additional file 2). This was the case for genes involved in the plant response to DNA damage stimulus, particularly DNA repair, and those involved in the regulation of hydrolase activity. A significant over-representation of differentially regulated genes classified as ‘regulation of defense response’ was also found in ‘Président Roulin’. Again, no under-representation of any biological pathways was found.

### Quantitative real-time reverse transcriptase PCR

To validate the reliability of our cDNA-AFLP analysis, qRT-PCR was performed on 24 pathogen-responsive TDFs representative of almost all functional categories identified, with a preference for defense-related genes and genes possibly involved in pathogenesis. Table 3 presents separately the qRT-PCR results carried out on the same RNA samples used for the cDNA-AFLP analysis and on a biological replication of the experiment. Results were expressed as fold-change of ‘Président Roulin’ (partially resistant) and ‘Gala’ (susceptible) after pathogen attack, in respect to mock-inoculated leaves. In general, expression data provided by qRT-PCR were in good agreement with profiles detected by cDNA-AFLP. When performed on RNA samples used for cDNA-AFLP, qRT-PCR confirmed the pattern of gene expression of 20 TDFs (83%). Expression of 22 of these RNA samples was then further verified by qRT-PCR on a biological replicate of the experiments, and the pattern of expression of 13 of these samples was confirmed (59%). TDFs that were not in accordance with the cDNA-AFLP showed no significant changes in expression in ‘Président Roulin’ (i.e. 42BUHcrVf, 43CU118, 39 AU13). For three TDFs, strong changes in gene expression (more than five-fold) were detected in infected leaves of ‘Président Roulin’ in both experiments (51HU129’, 44GU182 and 44EU122). Two different TDFs (43DU149 and 43DU149’) cloned from the same band and showing a significant increase in intensity after pathogen challenge were both confirmed to be up-regulated after pathogen attack. In most cases, no significant changes of expression were detected in infected leaves of the susceptible ‘Gala’ cultivar.

### Co-localization of the TDFs with RGA, QTL or apple scab major R genes

Approximatively, 40% of the TDFs anchored *in silico* on one of the 17 chromosomes of apple were localized in the proximity (within 250 kb) of RGAs clusters, QTLs or major R genes for apple scab resistance (Table 4). Nevertheless, this frequency was not significantly greater than those calculated for random ESTs derived from an uninfected apple leaves library [34]. So co-localization could have occurred purely by chance. However, considering separately the three classes of TDFs (group I, IIa and IIb), we found that group I and IIb of TDFs mapped at a greater frequency in the vicinity of major R genes than EST from AELA library (5% instead of 1%).

**Table 4:** **Frequency of co-localization of TDFs from ‘Président Roulin’ with RGAs, QTLs and major apple scab R genes**

Mapped	Number ^b^	Cluster RGA ^c^ (%)	QTL ^d^ (%)	Major R gene ^d^ (%)	QTL/cluster RGA/major gene (%)
TDFs^a^	387	22	21	4**	40
TDFs group I	130	24	17	5***	38
TDFs group IIa	110	22	24	4	38
TDFs group IIb	147	22	22	5**	44
Uninfected apple library AELA	501	20	21	1	38

^a^Transcript-derived fragments (TDFs) at 48 hours after inoculation by *V. inaequalis*: genotype-specific TDFs (group I), pathogen-responsive TDFs expressed in common by both genotypes (group IIa) and pathogen-responsive and genotype specific TDFs (group IIb). Frequencies of co-localization of TDFs were compared to frequencies observed with an uninfected apple library AELA [34] using a χ^2^ test. P values are indicated as follows: ***= P <0.001, **= 0.001 < P <0.01, and *= 0.01 < P <0.05.

^b^Number of cDNA mapping onto the apple chromosome with a threshold E-value < 0.001 by BLAST analysis against homologous apple genomic sequences with known chromosomal locations [104].

^c^clusters of resistance gene analogues (RGAs) [94].

^d^Quantitative trait loci (QTLs) and major R genes for apple scab resistance [3, 13, 14].

## Discussion

Plant disease resistance and susceptibility are governed by the combined host and pathogen genotypes, and depend on a complex exchange of signals and responses occurring under given environmental conditions. During the long process of host-pathogen co-evolution, plants have developed various elaborate mechanisms to ward off pathogen attack [40]. In addition to constitutive defense, it has been postulated that a key difference between resistant and susceptible plants is the timely recognition of the invading pathogen, and the rapid and effective activation of host defense mechanisms. Such induced resistance mechanisms have been demonstrated at transcriptional level in numerous studies on plant-pathogen interactions involving either complete [26, 41–44] or partial disease resistance [45, 46]. In both types of resistance, pathogen attack was accompanied by activation of host plant response genes and accumulation of corresponding gene products. Based on these findings, our cDNA-AFLP study attempted to elucidate the molecular mechanisms underlying partial resistance against apple scab by the identification of differentially expressed transcripts between ‘Président Roulin’ (partially resistant) and ‘Gala’ (susceptible) after pathogen attack.

Effective identification of differentially expressed transcripts after scab infection requires the determination of the stage of pathogen development at which it is first affected by the host’s defense mechanisms. This is important particularly in investigations built upon the hypothesis that resistance involves the induction of defense pathways. To identify the appropriate time-point for the extraction of RNA, we compared the kinetic of pathogen development on leaves of ‘Président Roulin’ (partially resistant) and ‘Gala’ (susceptible). Light microscopic assessment identified 48 hpi as the critical time-point when the pathogen is first affected by the host’s responses during formation of appressoria and stroma. In fact, whereas the early stages of pathogen growth occurred at the same rate in both cultivars, significant differences were observed in later stages, when development of subcuticular stroma subsequent to cuticle penetration was reduced by greater extent in the partially resistant cultivar compared to the susceptible one (Figure 1). These differences are concordant with the literature with various susceptible and resistant apple cultivars [47, 48] and led to a reduced disease severity and sporulation on ‘Président Roulin’ 21 days after infection. This also suggests that no particular pre-formed chemical or structural defense barriers are present at the leaf surface of ‘Président Roulin’. The choice of 48 hpi as the key time for our gene expression study is further motivated by the fact that in various plant-pathogen interactions the accumulation of defense-related transcripts in the host is often concomitant to the formation of appressoria and the development of the intracellular hyphae [49–52]. When this cytological event occurs, the molecular dialog between the pathogen and the host presumably begins. The host detects the pathogen and initiates defense-related transcriptional responses [53].

As for any transcriptome study, a genome-wide screen for differentially expressed genes based on cDNA-AFLP requires that as many transcripts as possible are analyzed in a unique way, and that the data obtained are informative enough to allow characterization of the transcripts. Considering criteria such as the genomic region of the restriction sites (coding or non-coding region), the redundancy and the length of the TDFs being produced, we showed *in silico* that the restriction enzyme pair *Eco*RI/*Mse*I was the most appropriate for our cDNA-AFLP analysis. This restriction enzyme combination was successfully used in similar experiments on apple [25, 26, 32, 54, 55]. In our study, this enzyme combination yielded a very high informative content (69% of the TDFs could be effectively annotated) with limited redundancy (sequence assembly yielded only 29 contigs out of the 536 unclustered sequences).

The cDNA-AFLP method has the advantage that all treatments, time points and genotypes under investigation can be concurrently compared [56, 57]. In our study, we estimated that we analyzed a representative sample of approximatively 19% of the genes expressed in the leaves of ‘Président Roulin’ and ‘Gala’ in response to *V. inaequalis* infection at 48 hpi. Our estimation corroborates with the data from the recent sequencing of the apple genome project since our 10,250 bands would represent about 18% of the 57,386 putative genes in apple [38]. We highlighted that the differences between partially resistant and susceptible responses to *V. inaequalis*, were more quantitative (different regulation of expression of the same genes in both cultivars) than qualitative (different genes being expressed in both cultivars), and that quantitative differences mainly involved up-regulation of genes (71% of up-regulations). As similarly demonstrated in the literature [58, 59], we can argue that, despite the very clear phenotypic differences observed between the partially resistant and susceptible responses, the timing and amplitude of gene induction could have a greater influence on the disease resistance status of the plant than the identity of genes activated. Under-representation of down-regulated TDFs has also been reported in apple - *V. inaequalis* interaction [25] and in other pathosystems [60–62] but it has been suggested that such under-representation could be mainly due to the two-step PCR of the cDNA-AFLP method [61]. The overall similarity of transcriptional signature between ‘Président Roulin’ and ‘Gala’ is further demonstrated by the observation that genes were regulated in the same direction in both cultivars: when a TDF was up-regulated in one cultivar, up-regulation was also observed in the other one (and inversely) (Figure 4). Finally, all ‘Président Roulin’ pathogen-responsive TDFs were also found to be expressed in the mock-control from high, to intermediate or low levels, supporting the idea that many genes involved in the defense reaction were already expressed in resistant genotype before pathogen challenge [26, 63].

The potential presence of multiple sequences in one band cut from the gel is the major disadvantage of cDNA-AFLP fingerprinting. In our study, this mis-cloning has been observed for 38% of the excised bands and could be an underestimate of the multiplicity of sequences per band since only two clones per band were analyzed. Each restriction enzyme pair may in fact result in different fragments of the same size which are visualized as a single band on a cDNA-AFLP gel autoradiography. Therefore, confirmation of gene expression by an independent technique is required, as for hybridization-based assay like microarray. In this study, expression profiles observed by cDNA-AFLP were confirmed by qRT-PCR in 83% of the 24 TDFs under examination (on the same cDNA samples used for the AFLP, Table 3). Results were similar to those reported by Baldo et al. (79% of 28 ESTs) [55] and Paris et al. (81% of 32 TDFs) [26]. The percentage of confirmation decreased when using an independent biological replication (59%). According to these results it can be assumed that the modulation of about 83% of the observed TDFs is confirmed; however extending data to at least a biological repetition is highly recommended to further confirm that gene modulation strictly depends on the biological system under study (i.e. the plant-pathogen interaction). This consideration is valid not only for cDNA-AFLP but for all transcriptional analysis as modulation of gene expression can be influenced by other factors (environment, biotic stresses…). However, in our opinion, two or more sequences from one band can contribute to the change of band intensity observed on the autoradiography, as suggested by the fact that two different sequences cloned from the same band were both confirmed to be up-regulated after pathogen attack (43DU149 and 43DU149’).

The GO analysis of differentially expressed TDFs revealed that they are represented by a high diversity of functional categories (Figure 5). This is not surprising since, with the emerging of genome-wide gene expression profiling technologies, it is now clear that plant response to pathogens is associated with massive changes in gene expression. For example, in an *Arabidopsis* microarray, more than 2000 genes (out of 8000 genes) involved in a broad range of biological responses were regulated in response to the bacterial pathogen *Pseudomonas syringae*[64]. In our study, the annotation of ‘Président Roulin’ pathogen-responsive TDFs indicates that they may act in early events of plant defense response such as pathogen recognition (e.g. some TDFs encoded for putative nucleotide-binding site leucine-rich repeat (NBS-LRR) proteins [65]), or in signal transduction (e.g., mitogen-activated protein (MAP) kinase [66]). Other TDFs identified may play a role in the later stages of the ‘Président Roulin’ defense response with the induction of genes aiming to stop or reduce the invasion of the host by pathogens. In plants, this often leads to the hypersensitive response, a form of localized programmed cell death orchestrated by the oxidative burst. TDFs involved in such reactions encoded for gluthatione-S-transferase [67], peroxidase [68], E3 ubiquitin protein ligase [69] and cysteine protease/cysteine protease inhibitor [70].

In order to understand the global molecular pathways involved in partial resistance, gene enrichment analysis were conducted to statistically determine whether specific GO terms were enriched in different sets of cDNA. First we demonstrated that the same functional categories were involved in our up- and down-regulated set of TDFs (group II + and II -). These results differ from the findings of Paris et al. [26] who found that TDFs similar to genes putatively involved in defense responses were generally up-regulated in R gene-mediated resistance (*Rvi6*/*HcrVf2*), while those putatively involved in general metabolism were down-regulated. In contrast, we demonstrated that genes involved in stress response (including disease/defense response) and in photosynthesis were preferentially regulated in the partially resistant cultivar ‘Président Roulin’ after pathogen attack, as compared to two published EST libraries from *Malus* x *domestica* uninfected young expanding leaves [34, 35] (Figure 6). When stress response genes could participate in the partial resistance of ‘Président Roulin’ against *V. inaequalis*, regulation of genes of the photosynthetic pathway might be the first step towards the appearance of chlorotic spots on infected leaves, which in the apple - *V. inaequalis* incompatible interaction appear about 8–12 days after inoculation [25]. It is also well known that defense responses is energy intensive [73] and requires transcriptional activation of genes [71]. These could be the reason why we found in ‘Président Roulin’ an over-representation of genes involved in different catabolic/metabolic processes, that might ‘fuel’ the implementation of downstream defense response, and in the regulation of gene expression. Nevertheless, as these later GO categories appear to be significantly over-represented in only one of the two libraries being compared, their involvement in the resistance of ‘Président Roulin’ against *V. inaequalis* still has to be confirmed. Finally, our comparison between the ‘Président Roulin’ cDNA library with the completely resistant *Rvi6* (*HcrVf2*)-transformed ‘Gala’ lines [25] revealed both similar events (e.g. transport, photosynthesis functions), and differences in some biological process categories (Figure 7). Among the differences (Additional file 2) we noticed an over-representation in ‘Président Roulin’ of genes involved in (1) the regulation of defense response (e.g. E3 sumo ligase SIZ1 proteins [72] and tocopherol cyclase [74, 75]; (2) proteins involved in the regulation of hydrolase activity, also recognized to be key enzymes in the regulation of programmed cell death in incompatible plant-pathogen interactions (e.g. cysteine protease inhibitor and a 13-fold repressed cysteine protease [70, 76, 77]); (3) proteins involved in the response to DNA damage stimuli. This latter category is thought to be involved in the plant response to abiotic stress such as UV-B [78] and osmotic stress [79], but to our knowledge, these proteins were not yet known to be involved in quantitative disease resistance.

Beside these slight differences between complete and partial resistance, large parts of the transcriptional signatures did not demonstrate enrichment for genes in particular functional categories. This finding is consistent with the hypothesis that partial resistance could be due in part to the same genes governing complete resistance. To illustrate this hypothesis, some classical R genes encoding for the NBS-LRR family protein were found to be up-regulated in the partially resistant cultivar ‘Président Roulin’ after pathogen attack. In that context, partial resistance could be due to defective R genes that recognize with low efficiency pathogens and trigger weak defense response [65]. This may result either from mutation in the R genes themselves or at the corresponding avirulence locus of the pathogen. In fact, there are compelling lines of evidence that allelic variants of R genes account for quantitative disease resistance in plants (e.g. *Xa21* in rice-blast interaction [80]). In the same way, when a pathogen strain overcomes an R gene due to a mutation at the avirulence locus, it has been proved that the “defeated” R gene still has a residual effect and can act as a QTL against virulent strains of the pathogen. This phenomenon has been observed for the major resistance genes *Rvi4* (*Vh4*) [3] and *Rvi6* (*Vf*) [81] in the *V. inaequalis* - apple interaction, and also in rice bacterial blight [82], wheat stem rust [83] and powdery mildew pathosystems [84]. Likewise, in the same way as major resistance genes, several QTLs have been shown to be isolate-specific [12, 85, 86] and co-localization of QTLs and R genes has been noted in several species including apple [62, 87–89]. Another evidence suggesting that genes controlling partial resistance in ‘Président Roulin’ could share structural and functional similarities with R genes resides in the fact that some subsets of our cDNA library mapped at greater frequency in apple genomic regions known to carry major scab R genes [3, 14] (as compared to random EST from uninfected apple library). The co-localization of ESTs with genomic regions carrying disease resistance factors (R genes, QTLs or RGAs) has been already reported in various genome-wide analyses studies [90–93]. No significant co-localization of our cDNA library with apple scab QTLs [3, 13, 14] nor apple RGAs [94] has been found. Obviously, we could only check for co-localizations with R genes and QTLs that have been detected so far. Moreover, information on the genomic loci that can regulate the expression level of the TDF of interest is still lacking from this analysis. In fact, the measured mRNA levels can either be the product of regulation of the parent gene or of another gene, mapping somewhere else in the genome (cis- or trans- regulatory elements) [95].

## Conclusions

In conclusion, this study provides a wide transcriptional profile analysis for the comprehension of key events in partial resistance of ‘Président Roulin’ and highlights possible candidate resistance genes. We found altered gene expression in resistant and susceptible plants in response to *V. inaequalis* that involved many functional categories. Genes acting in pathogen recognition (NBS-LRR) as well functioning downstream of the initiated defense signaling pathways were identified. Biological processes related to stress response and photosynthesis were found to be over-represented in infected leaves of the partially resistant cultivar compared with two published libraries of uninfected apple leaves. In addition, through comparison between partial and complete resistance, the pathogen-responsive cDNA library revealed common physiological events, but differences in regulation of defense response, in the regulation of hydrolase activity, and in response to DNA damage stimuli. Finally, TDFs from ‘Président Roulin’ mapped more frequently in the vicinity of major R genes for apple scab resistance, suggesting that quantitative and complete resistance could be governed by the same types of genes. A functional analysis of the differentially expressed genes will allow more insights into their possible role in the quantitative resistance reaction of ‘Président Roulin’ against *V. inaequalis*. For example, an assessment of the differential expression of candidate resistance genes over different time points after infection could be investigated to find out how resistance is regulated by quantitative and/or kinetic enhancements. Also, analysis of candidate gene expression data in a segregating population could infer causal relationship between the differential expression of the genes and the resistance phenotype of the progeny. Finally, these candidate resistance genes might be at the basis of the development of molecular marker tools to be used in a genome-informed breeding program to speed-up the selection process of resistant plants.

## Methods

### Plant material and inoculation with *Venturia inaequalis*

Plants of the partially resistant Belgian cultivar ‘Président Roulin’ and the susceptible cultivar ‘Gala’ were grafted on M9 rootstocks and grown in a greenhouse at 20°C under 16 hours of illumination by daylight-incandescent lights. In the frame of the DARE European project, ‘Président Roulin’ has been shown to be resistant to a large range of inocula, including local mix inocula and monoconidal *V. inaequalis* strains belonging to the race 1, 6, 7 [96] and 2, 8, 9 (data not showed).

In this study we used six monoconidial strains of race 1 *V. inaequalis* originating from the INRA collection at Angers, France (EU-B04, EU-B16, EU-D49, EU-F05, EU-F11 and EU-I09) to prepare the inoculum. Each strain, first grown in Petri dishes for 10 days on malt extract agar and covered by a cellophane membrane, was multiplied separately on young seedlings raised from open-pollinated ‘Golden Delicious’. Infected leaves were dried and stored at −20°C for not more than 1 year before use. Conidia were harvested by shaking the leaves in sterile water. A conidial suspension was prepared by mixing conidia of the 6 strains at a final concentration of 2.5 × 10^5^ conidia per milliliter. The conidial suspension was sprayed onto young leaves of actively growing ‘Gala’ and ‘Président Roulin’ plants in quantities sufficient to form small droplets on the leaf surface before run off. The inoculated plants were incubated at 20°C under maximum relative humidity (RH) for two days and were then transferred to the greenhouse (20°C, 60-80% RH). Control plants were inoculated with sterile water. Conidia vitality was verified after 24 h by determining the fraction of germinated conidia (~70% for all the isolates). Levels of scab infection were scored for each plant 21 days after inoculation. To reduce the level of biological variation among samples, two plants per treatment were used. For each plant, the three youngest leaves were collected at 48 hpi, immediately frozen in liquid nitrogen and stored at −80°C until RNA extraction.

### Microscopic investigation of fungal development

To identify the optimal timing of sampling for the subsequent cDNA-AFLP experiment, progress of pathogen development was followed on ‘Président Roulin’ and ‘Gala’ using light microscopy. Plants were spray-inoculated and incubated as described above. At 1, 4, 6, 16, 20, 24, 32, 48, 54 and 120 hpi, the youngest expanding leaf of each cultivar was sampled (one leaf/sampling time/cultivar), cleared overnight in 99% methanol and stained with periodic acid-basic fuchsine according to the method by Preece [97]. Samples were thoroughly rinsed with water and mounted onto glass slides. Pathogen development stages were examined at the different time points by observing at least 200 conidia per sampling time for each of the cultivars.

### *In silico*cDNA-AFLP simulations

A total of 450 full-length apple cDNAs sequences from the study by Newcomb et al. [34] were analyzed with the AFLPinSilico program [98]. The combinations of restriction enzymes used here were *Sau3*A, *Taq*I, *Dde*I, *EcoR*I, *EcoR*II, and *Apo*I as first sites in combination with the restriction enzyme *Mse*I as second site, and *vice-versa*. The following parameters were estimated: percentage of cleaved cDNA, percentage of cDNA visualized on a gel, number of cleavage sites per cDNA, average distance between the last recognition site and the polyadenylation site, and the mean size of the restriction fragments.

### RNA extraction and cDNA-AFLP

Total RNA was isolated from 100 mg of leaf material collected at 48 hpi (two plants per treatment), using the extraction method described by Gasic et al. [99]. After DNase I treatment (Fermentas Inc), purification of mRNA was performed starting from 250 μg total RNA using the Qiagen Oligotex mRNA kit (Qiagen Inc.). RNA purity and concentration was measured with the Nanodrop technology (Thermo Scientific Inc.). Double stranded cDNA was finally obtained starting from 500 ng mRNA following the instructions of the Superscript Double Stranded cDNA Synthesis kit (Invitrogen Inc.).

cDNA-AFLP analysis was performed with the AFLP Core Reagent kit (Invitrogen Inc.) as recommended by the manufacturer. The double-stranded cDNA was digested with *EcoR*I and *Mse*I and ligated to the corresponding *EcoR*I and *Mse*I adapters. The pre-amplification step was carried out using 20 cycles of amplification (94°C for 30 s; 56°C for 1 min; 72°C for 1 min) starting from a 5 μl aliquot of a 1:2 dilution of the ligation reaction and 75 ng of primer corresponding to the *Mse*I and *EcoR*I adapter sequence without any extension, in 50 μl total volume. After 10-fold dilution of the PCR fragments, specific amplifications were carried out with a total of 141 *EcoR*I and *Mse*I primer combinations containing two (123 primer combinations with EcoRI + 2/MseI + 2), or three additional selective bases at the 3’ end (18 primer combinations with EcoRI + 2/MseI + 3, EcoRI + 3/MseI + 3 or EcoRI + 3/MseI + 1). The *Eco* primers were labeled with [γ33P] dATP. Amplification products were separated by electrophoresis at 60 W on a vertical denaturing polyacrylamide gel (5%) containing 7 M urea for 3 hours 30 minutes. Gels were transferred onto Whatman 3MM paper before drying. Repeatability of the technique was checked trough an experimental replication of the selective amplification step (for a few selective primer pairs) starting from the same pre-amplified cDNA samples.

Bands corresponding to the TDFs were visualized on the polyacrylamide gel by autoradiography using X-ray films. Band intensities were digitized using the PhosphorImager tool (Biorad) and were quantified using QuantityOne software (Biorad). For each primer pair combination tested, only cDNA-AFLP profiles with the same global band intensity among genotypes and treatments were compared. This is presumed to reflect the fact that equivalent amounts of amplified cDNA were compared. We considered all the TDFs whose expression ratio between inoculated and non-inoculated control treatments was above the threshold of two (fold-change >2) as significantly up-regulated, and all those whose ratio was below the inverse threshold (fold-change <1/2) as significantly down-regulated.

Bands of interest belonging to the cultivar ‘Président Roulin’ were cut from the polyacrylamide gels, eluted in 50 μl of water, re-amplified with the selective primers and cloned into Pjet 1.2 vector (Fermentas). Two transformed colonies per TDF with an insert of the expected size were subsequently sequenced (Macrogen Inc.). cDNA sequences were manually checked for overall quality, and vector and primer contaminations were trimmed.

### Bioinformatics analysis

In order to limit redundancy and to produce a longer consensus sequence, the sequences were clustered using Egassembler [36, 37]. We then compared the contigs and singletons identified with the whole apple genome sequence assembly v1.0 [38] by BLAST sequence similarity search. This second step allowed us to identify the TDFs that originated from the same original contig (and thus from the same gene) of the apple genome assembly. The functional annotation of this non-redundant database was performed using the automatic Blast2GO bioinformatics tool [100]. Basically, input sequences were queried by BLAST-X against the GenBank non-redundant sequences and ESTs database at the National Center for Biotechnology Information (NCBI) database, taking similarities with an E-value < 10^−3^ as significant matches. Then, the program extracts the GO terms associated to each of the obtained hits and returns an evaluated GO annotation for the query sequences (E-value < 1e^−6^). These E-values were the default values proposed by Blast2GO.

In order to detect GO annotations whose abundance was significantly different between different sets of annotated genes, a gene function enrichment analysis was performed using Blast2GO [100]. This software employs Fisher’s exact test to estimate the significance of associations between two categorical variables using a single test p-value. A set of GO terms that are under- or over-represented at a specified significance value (P < 0.05) were obtained as a result of performing the enrichment analysis. For this analysis, two subsets of our cDNA-AFLP library were compared: the TDFs being up- and down-regulated in ‘Président Roulin’ after pathogen infection (group II + and II -). We also compared our cDNA-AFLP library (group I and group II) from infected leaves of ‘Président Roulin’ to: (1) two EST libraries from uninfected young expanding leaves of *Malus* × *domestica* ‘Royal Gala’ (AELA) [34] and ‘Wijcik’ (Mdstw) [35], and (2) a cDNA library [25] of differentially expressed transcripts from *Rvi6* (*HcrVf2*)-resistant transgenic ‘Gala’ lines challenged by *V. inaequalis* (obtained by suppression subtractive hybridization between *Rvi6* (*HcrVf2*)-transformed ‘Gala’ lines and susceptible ‘Gala’ plants). The last three libraries were assembled in contigs and singletons using the EGassembler tool [37] before performing the analysis. Sequences were annotated with the bioinformatics tool Blast2GO using the same parameters as those applied for the annotation of the cDNA-AFLP fragments.

### Quantitative real-time reverse transcription PCR (qRT-PCR)

qRT-PCR was carried out on the same RNA samples used for the cDNA-AFLP analysis and on RNA derived from one independent biological replication of the experiment. Specific TDF primers were designed with the software Primer3 [101] and qRT-PCR was performed using Biorad CFX96 and Maxima SYBR Green qPCR master mix (Fermentas Inc.), following the instruction of the manufacturer. A list of the specific primer pairs used for each TDF and product lengths is given in Additional file 3. PCR conditions were the same for all primer pairs: initial denaturation at 95°C for 10’ followed by 40 cycles of denaturation at 95°C for 15”, annealing at 60°C for 30” and extension at 72°C for 30”. Fluorescence data were collected at the end of the annealing step. Following cycling, samples were denatured at 95°C for 10”. The melting curve was analyzed to differentiate between the desired amplicons and any primer dimers or DNA contaminants (in the range 65°–95°C, with a temperature increment of 0.5°C for 5″). Each reaction was run in duplicate. The LinRegPCR software was used to confirm that individual PCR efficiencies were about 2 for each primer pair [102]. The relative expression ratio of the target genes between scab-inoculated and water-treated plants was evaluated using the ΔΔCt method described by Applied Biosystems (Relative expression ratio = 2^ΔΔCt^), with the glyceraldehyde 3-phosphate dehydrogenase gene (*GAPDH*) as the internal reference (primers sequence F-5′CAAGGTCATCCATGACAACTTTG3′, R-5′ GTCCACCACCCTGTTGCTGTAG3′). In fact, as it was the case in other qRT-PCR studies conducted on apple [33, 103], the *GAPDH* gene appeared to be the best housekeeping gene in our experimental conditions. Contrary to the elongation factor gene (*EF,* primers sequence F-5′TACTGGAACATCACAGGCTGAC3′, R-5′TGGACCTCTCAATCATGTTGTC3′), expression of the *GAPDH* was stable in scab-inoculated and water-treated leaf samples (Additional file 4). Individual relative expression values were then subjected to the ANOVA procedure, using Minitab 16 software, at a statistical significance level of P < 0.01.

### *In Silico*mapping and co-localization of the TDFs with RGAs, QTL or apple scab major R genes

TDFs sequences were searched by BLAST-N within the whole genome sequence assembly v1.0 of apple [38] on the Genome Database of Rosaceae (GDR) [104] taking into account the best BLAST result (E-value < 0.001). Clusters of apple resistance genes analogues (RGAs) [90], QTLs and major scab resistance genes already identified in apple [3, 13, 14] were also anchored *in silico* to the apple genome sequence. Molecular markers flanking the major scab R genes and QTLs were obtained from the HIDRAS (High-quality Disease Resistant Apples for Sustainable Agriculture) website [105] and searched in the apple genome sequence by BLAST-N. Only SSR markers having an E-value ≤3e-3, a ratio of matched bases to marker sequence equal to 100% and a position on the expected chromosome on the ‘Golden Delicious genome sequence assembly (according to their genetic position) were anchored to the physical map. Clusters of RGAs and their physical position on the ‘Golden delicious’ genome assembly were retrieved from the publication of Perazzolli et al., [90]. Only TDFs mapping inside a QTL confidence interval or mapping within 250 kb from any cluster of RGAs or major scab R gene were considered to co-localize in the genomic regions involved in resistance. This distance has been used in previous publication as the average distance separating genes inside a cluster [90]. In order to determine if TDFs co-localized only by chance, frequencies of co-localization were compared to frequencies observed for ESTs from an uninfected apple library [34] using a χ2 test employing the statistical software Minitab 16.

## Availability of supporting data

The whole cDNA-AFLP library isolated from ‘Président Roulin’ has been deposited at DDBJ/EMBL/GenBank under the accession numbers JZ719314 to JZ719813. Other supporting data are included as Additional files 1, 2, 3 and 4.

## Electronic supplementary material

Additional file 1: **Sequence annotation using the bioinformatics software Blast2GO.** This Table provides a full list of differentially expressed TDFs isolated from ‘Président Roulin’, their expression pattern according to the cDNA-AFLP and their annotation using the bioinformatics software Blast2GO. (XLSX 101 KB)

Additional file 2: **Over-representation of GO categories in our cDNA-AFLP library compared to** ***Rvi6*** (***HcrVf2*** **)-’Gala’ transformed library **[25]. This table provides a list of TDFs and their annotation belonging to Biological Process GO categories that were over-represented in the ‘Président Roulin’ cDNA-AFLP library (partially resistant) compared with a cDNA library from completely resistant *Rvi6* (*HcrVf2*)-transformed ‘Gala’ lines challenged with *V. inaequalis*[25]. Gene enrichment analysis was conducted with the software Blast2Go using Fisher’s Exact Test at a p-value <0.05. (XLSX 13 KB)

Additional file 3: **List of primer pairs used for qRT-PCR validation of differential gene expression revealed by cDNA-AFLP.** This table provides the primer sequences used for the qRT-PCR analyses. (XLSX 13 KB)

Additional file 4: **Expression variation of candidate housekeeping genes in apple leaves challenged by** ***V. inaequalis*** **and mock-inoculated.** This figure shows that the *GAPDH* gene appeared to be the best housekeeping gene in our experimental conditions. Contrary to the *EF* gene, expression of the *GAPDH* was stable in scab-inoculated and water-treated leaf samples. (DOCX 29 KB)
