# Supplementary material for: Gene expression profiling by cDNA-AFLP reveals potential candidate genes for partial resistance of ‘Président Roulin’ against Venturia inaequalis
Source: BMC Genomics. 2014 Nov 29;15:1043. doi: 10.1186/1471-2164-15-1043 (PMC4302150; doi:10.1186/1471-2164-15-1043)
Supplement: Supplementary file 4 — Additional file 4: Expression variation of candidate housekeeping genes in apple leaves challenged by V. inaequalis and mock-inoculated. This figure shows that the GAPDH gene appeared to be the best housekeeping gene in our experimental conditions. Contrary to the EF gene, expression of the GAPDH was stable in scab-inoculated and water-treated leaf samples. (DOCX 29 KB) [file 12864_2014_6851_MOESM4_ESM.docx]

**Additional File4: Expression variation of candidate housekeeping genes in apple leaves challenged by *V. inaequalis* and mock-inoculated.** Two housekeeping genes have been tested on 'Président Roulin' and 'Gala' leaves: glyceraldehyde 3-phosphate dehydrogenase (*GAPDH*) and elongation factor gene (*EF*). For each cultivar and each gene, boxes represent lower and upper quartiles of cycle thresholds range obtained in inoculated (2 qRT-PCR technical replications) and control mock-inoculated (2 qRT-PCR technical replications) plants, with medians indicated. . Whiskers represent 10th and 90th percentiles. Graph was plotted using Minitab 16.0.
